# Hydrogen Exchange through Hydrogen Bonding between Methanol and Water in the Adsorbed State on Cu(111)

**DOI:** 10.1021/acs.jpclett.3c00161

**Published:** 2023-03-08

**Authors:** Roey Ben David, Adva Ben Yaacov, Baran Eren

**Affiliations:** † Department of Chemical and Biological Physics, Weizmann Institute of Science, 234 Herzl Street, 76100 Rehovot, Israel

## Abstract

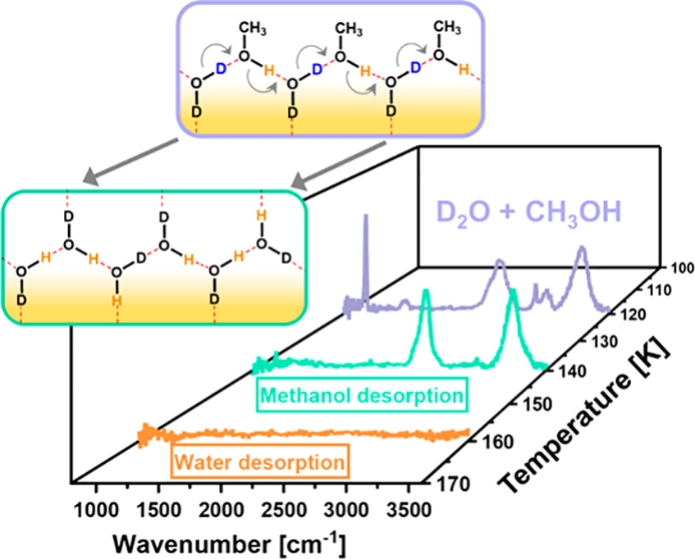

The interaction between submonolayers of methanol and water on Cu(111) is studied at 95–160 K temperature range with surface-sensitive infrared spectroscopy using isotopically labeled molecules. The initial interaction of methanol with the preadsorbed amorphous solid water at 95 K is through hydrogen-bonding with the dangling hydroxyl groups of water. Upon increasing the temperature up to 140 K, methanol and deuterated water form H-bonded structures which allow hydrogen–deuterium exchange between the hydroxyl group of methanol and the deuterated water. The evolution of the O–D and O–H stretching bands indicate that the hydrogen transfer is dominant at around 120–130 K, slightly below the desorption temperature of methanol. Above 140 K, methanol desorbs and a mixture of hydrogen-related water isotopologues remains on the surface. The isotopic composition of this mixture versus the initial D_2_O:CH_3_OH ratio supports a potential exchange mechanism via hydrogen hopping between alternating methanol and water molecules in a hydrogen-bonded network.

Methanol steam reforming (MSR) reaction is a promising option for onboard hydrogen generation from a mixture of methanol and water vapors via a heterogeneous catalytic reaction. Cu-based materials are among the catalysts with highest potential for this reaction thanks to their high activity at relatively low operating temperature (200–300 °C).^1,2^ The first step of the MSR reaction likely involves the molecular adsorption of both methanol and water, which are two hydrogen-bond (H-bond) forming molecules; thus, lateral interactions between them might play a crucial role. This molecular adsorption step usually has a relatively short lifetime under realistic reaction conditions of temperature and pressure; however, it may determine the nature of the initial surface intermediates and thereby steer the following reaction pathway. A poignant illustration of the importance of molecular precursors in dissociative adsorption is methanol on Cu surfaces, which was discussed in one of our recent studies.^3^

Two fundamental aspects of the interaction between methanol and water on metallic surfaces are the H-bonded structures and the hydrogen-atom transfer between the adsorbed molecules. Principally, the H-bonded structures and the adsorption geometry are derived from the energy balance between intermolecular interactions and the interaction with the surface.^4,5^ As the mechanism of the hydrogen-atom transfer is strongly related to the H-bonded structures, it could also be affected by the underlying substrate, especially for the first monolayer.

Detailed atomic and molecular level characterization of the H-bonded structures formed on surfaces is a challenging task, which typically requires the combination of high-resolution real and reciprocal space imaging tools (such as scanning tunneling microscopy (STM), atomic force microscopy (AFM), low energy electron diffractometry (LEED), etc.) together with theoretical calculations (such as density functional theory (DFT), molecular dynamics (MD), etc.).^5−8^ The H-bonded structures of methanol–water clusters have been barely studied in the literature with these methods. A recent study,^9^ which combined AFM and MD simulations on the interface of water–methanol solutions with graphite, revealed the formation of ordered interfacial H-bonded structures. These structures were identified as linear structures of an alternating H-bonded network of water and methanol. The H-bonded structures of pure water and pure methanol have been extensively studied. For instance, two H-bonded structures of methanol on Cu(111) were reported in the literature, which are linear chains and cyclic hexamers (i.e., rings of six methanol molecules).^10,11^ It was revealed by STM that linear chains form on the surface at low temperatures and high coverages and transform into hexamer clusters upon annealing. The thermally induced structural transformation was also observed in our previous work by polarization modulation–infrared reflection absorption spectroscopy (PM-IRRAS) and thermal desorption spectroscopy (TDS) measurements on Cu surfaces with different crystal orientations.^12^

For water adsorption on the Cu(111) surface, which is similar to other hydrophobic metal surfaces such as Au(111), a double bilayer model was suggested to describe the ordered structure.^7,13,14^ However, when water is dosed at low surface temperatures amorphous solid water (ASW) forms, which has an onset temperature for crystallization at ∼130–140 K.^13,15^ Mehlhorn et al.^13^ found that the double bilayer structure of water on Cu(111) might have various terminations, such as a faceted surface, pyramidal islands, and nanocrystallites, depending on the annealing temperature. At low surface temperatures (5–40 K) and at low coverages (in the order of ∼0.01 monolayers), water monomers and small H-bonded clusters can also form on Cu(111).^6,16,17^ The most basic unit of these clusters is a cyclic hexamer, i.e., a ring of six H-bonded water molecules, which is considered as the building block of the bilayer structure on hydrophobic surfaces such as Cu(111).

H-exchange between different water isotopologues or between water and alcohols in the adsorbed state has been investigated in several previous studies on different surfaces.^18−21^ The H–D exchange between H_2_O and D_2_O in amorphous and crystalline thin films of ice has been extensively studied in the context of astrochemistry of interstellar ice.^18,19,22−25^ It was found that the H–D exchange is thermally activated above ∼120 K and is accelerated at ∼150 K during crystallization.^18,23^ Lee et al.^18^ and Fisher et al.^24^ proposed various atomistic processes for the exchange mechanism. The activation energy for the H–D exchange in ASW was reported to be 32 ± 1 kJ/mol in a recent study of Lamberts et al.^22^ Similar to the H–D exchange in water, the H–D exchange between water and the hydroxyl group of methanol was found to take place above 120 K^21,26−28^ with a comparable activation energy (36 ± 7 kJ/mol^27^). Ratajczak et al. studied by Fourier transform IR (FTIR) spectroscopy the H–D exchange in ice films composed of 1–3% CD_3_OD in H_2_O, which were deposited on KBr at 90–110 K.^21^ By monitoring the O–D stretching band associated with HDO (∼2425 cm^–1^) versus temperature, they deduced a rapid exchange at 120 K and assumed proton transfer mechanism through the H-bonds. A similar exchange mechanism was also suggested for the H–D exchange between ethanol and water isotopologues coadsorbed on Au(111).^20^

In this work, we provide a rationale for the H-bonded structures of submonolayers of methanol and water on Cu(111), and what role they could be playing in the H–D exchange mechanism. Using PM-IRRAS, we follow the exchange of hydrogen atoms between the coadsorbed molecules as a function of temperature with the aid of isotope labeling. The evolution of the O–H and O–D vibrational bands between 90 and 160 K provided valuable piece of information about changes in the H-bonding, suggesting that a significant H-exchange between the OH group of regular methanol (CH_3_OH) and deuterated water (D_2_O) occurs at low temperatures, slightly below the desorption temperature of methanol in ultrahigh vacuum (UHV) conditions. Moreover, the difference between the desorption temperatures of methanol (∼140 K) and water (∼160 K) allowed us to capture the exchange product (mixture of water isotopologues, mostly HDO) on the surface. The isotopic composition of this product as a function of the initial D_2_O:CH_3_OH ratio is used to propose a model for the exchange mechanism and the H-bonding structure.

Figure 1 shows the evolution of the PM-IRRAS spectra after pure D_2_O adsorption (Figure 1a) and after coadsorption of D_2_O and CH_3_OH (Figure 1b) on the Cu(111) surface followed by increasing the temperature from 95 to 160 K. In both cases, 0.1 Langmuirs (1 L = 10^–6^ Torr·s) of each gas were dosed at 95 K, and D_2_O was dosed prior to methanol in the case of D_2_O + CH_3_OH. Using the kinetic theory of gases and assuming the sticking coefficient to be unity, exposure of 0.1 L is equivalent to a coverage of 0.02 monolayer (ML) of CH_3_OH and 0.025 ML of D_2_O. Table 1 summarizes the observed vibrational frequencies at 95 K and their assignments. In the pure D_2_O adsorption experiment (Figure 1a), the PM-IRRAS spectra at lower temperatures (95–130 K) are composed of two peaks: a small sharp peak at 2727 cm^–1^ and a broad asymmetric peak at ∼2530 cm^–1^. The small peak at 2727 cm^–1^ originates from the O–D stretch of dangling (non-H-bonded) OD groups of 3-coordinated D_2_O,^29−31^ while the broad peak corresponds to the vibrational features of H-bonded D_2_O molecules.^32,33^ The exact assignment of this band to the different vibrational modes is still controversial; however, it is generally viewed as a convolution of three vibrational bands: symmetric and asymmetric D-O–D stretching (labeled as ν_s_ and ν_a_, respectively), and overtone of the bending mode (2δ), which is enhanced by a Fermi resonance.^34,35^ The frequency order of ν_a_ > ν_s_ > 2δ is commonly accepted for these three overlapping bands. The characteristics of the H-bonded D_2_O band are sensitive to the H-bonding environment, the H-bonding structure, as well as the adsorption strength and geometry of the D_2_O molecules. In this particular case, the asymmetric peak at ∼2530 cm^–1^ obtained at low temperatures can be attributed to ASW clusters.^13,14,36^

**Figure 1:**
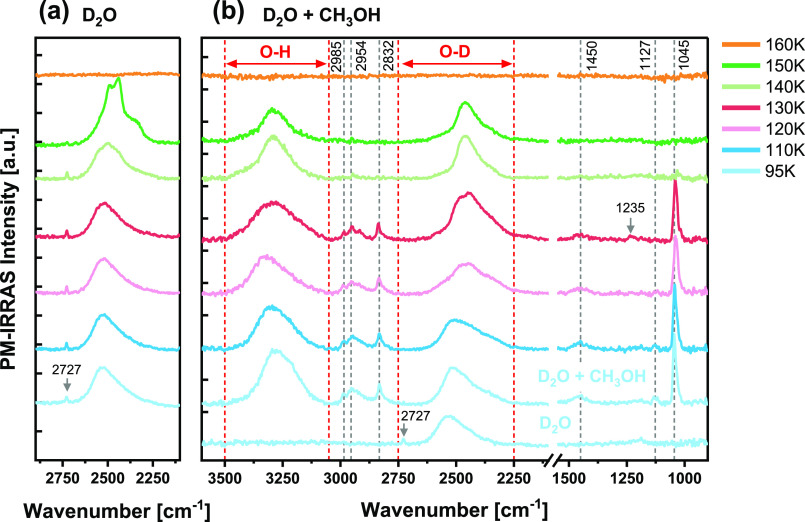
PM-IRRAS spectra of (a) D_2_O adsorption (0.1 L) and (b) D_2_O + CH_3_OH coadsorption (0.1 L of each, D_2_O dosed first) on a Cu(111) surface, followed by gradual annealing to various temperatures up to 160 K. Gray dashed lines indicate the vibrational bands of CH_3_OH, and their assignments are listed in Table 1.

**Table 1:** Summary of the Vibrational Frequencies (cm^–1^) Measured by PM-IRRAS for D_2_O Adsorption (0.1 L) and the Co-adsorption of D_2_O + CH_3_OH and D_2_O + CH_3_OD (0.1 L of each) on Cu(111) at 95 K^a^

	D_2_O (ASW)	D_2_O + CH_3_OH	D_2_O + CH_3_OD
ν(OH)	–	3278^m^	-
ν(OD)	2727, 2530	2513^w^	2515^w^, 2440^m^
2 × *a*″-ρ(CH_3_)	–	–	2476
ν_a_(CH_3_) + 2δ_a_(CH_3_)	–	2954, 2985	2916, 2954, 2987
ν_s_(CH_3_)	–	2832	2836
δ_s_(CH_3_) + δ_a_(CH_3_)	–	1450	1467
*a*″-ρ(CH_3_)	–	–	1232
*a′*-ρ(CH_3_)	–	1127	–
ν(CO)	–	1045	1040

a Key: ν, stretching mode; δ, bending mode; ρ, rocking mode; s, symmetric; a, asymmetric; *a*′ and *a*″, the notations of the two CH_3_ rocking modes; m, methanol-related peak; w, water-related peak. The assignments of the frequencies to the corresponding vibrational modes are based on refs (31 and 32) for D_2_O and refs (37 and 38) for CH_3_OH and CH_3_OD.

The PM-IRRAS spectra of D_2_O on Cu(111) remain unchanged after increasing the temperature up to 130 K (Figure 1a). At 140 K, slight changes occur in the shape of the H-bonded D_2_O band, which extends and shifts toward lower wavenumbers. Further heating to 150 K causes noticeable changes in the spectrum: the dangling O–D peak at 2727 cm^–1^ diminishes, and the H-bonded D_2_O band splits into two strong peaks at 2441 and 2489 cm^–1^ with a shoulder at ∼2350 cm^–1^. Heating to 160 K leads to a spectrum with no discernible features due to complete desorption of D_2_O from the surface. The changes in the vibrational band of D_2_O at 140–150 K, about 10 K below the desorption temperature in UHV, indicate a thermally activated rearrangement of the H-bonded structure. According to a previous high-resolution STM study, this structural transformation corresponds to a transition from amorphous to crystalline D_2_O ice.^13^ It was suggested that slightly below the desorption temperature the H-bonds are thermally activated and the water molecules are mobile enough to restructure into more stable and ordered H-bonded structures. A previous IR spectroscopy study on D_2_O adsorption on Pt(111) supports the assignment of the multipeak band at 150 K (Figure 1a) to an ordered double bilayer film of D_2_O.^36^ In the same study, following the growth of the second bilayer at 140 K, multiple peaks were detected at 2355, 2450, and 2489 cm^–1^, which are similar to the peak positions obtained in the current study.

The PM-IRRAS experiments on the D_2_O + CH_3_OH coadsorption can provide new insights on the intermolecular interactions between methanol and water in the adsorbed state. It can be seen in Figure 1b that following the addition of CH_3_OH (0.1 L) to the preadsorbed amorphous D_2_O layer (0.1 L) at 95 K, the dangling O–D peak of D_2_O (2727 cm^–1^) vanishes and the vibrational bands of methanol appear in the spectrum. The most prominent peaks of methanol are the C–O stretching, ν(C–O), at 1045 cm^–1^ and the broad O–H stretching peak, ν(O–H), at 3278 cm^–1^. Other bands are indicated by gray dashed lines in Figure 1b and their assignments are listed in Table 1. The disappearance of the dangling O–D peak is the first evidence that methanol interacts with the H-bonded D_2_O network by forming H-bonds with the ’free’ (uncoordinated) OD groups of adsorbed D_2_O. After methanol adsorption at 95 K, only minor changes are observed in the H-bonded D_2_O peak in the form of a slight redshift to 2513 cm^–1^. Further heating to 130 K results in further shifting and broadening of the O–D band, concurrent with broadening of the O–H band (∼3300 cm^–1^). At 140 K, the vibrational bands of methanol disappear due to methanol desorption, in agreement with the desorption temperature reported in our previous work for pure methanol on Cu(111).^12^ Interestingly, after methanol desorption both O–H (∼3290 cm^–1^) and O–D (∼2460 cm^–1^) bands remain in the PM-IRRAS spectra at 140–150 K, indicating a mixture of hydrogen-related water isotopologues, likely in the form of HDO as the main constituent. This is clearly a result of the hydrogen-atom exchange between the hydroxyl groups of methanol and that of deuterated water at low temperatures (*T* < 140 K). As a reference, the O–H and O–D stretching frequencies of HDO (diluted in D_2_O and H_2_O Ih-ice films, respectively) were reported to be 3279 and 2416 cm^–1^.^39^ The more or less equal intensities of the O–H and O–D bands at 140–150 K (Figure 1b) suggest a considerably high exchange ratio. In comparison, nearly similar intensities of O–H and O–D bands were previously reported for isotopically mixed ice nanoparticles with ∼51% OD.^32^ In line with previous studies,^20,21^ we suggest that the H-bonds are the channels through which hydrogen atoms can be transferred between water and methanol molecules in the adsorbed state. The different desorption temperatures of methanol (*T*_des_ ∼ 140 K) and water (*T*_des_ ∼ 160 K) allow us to capture the H–D exchange product on the surface at intermediate temperatures. Figure 2 schematically describes the evolution of adsorbed molecules on Cu(111) following D_2_O + CH_3_OH coadsorption at 95 K and gradual increase in surface temperature. In this model for *T* < 140 K (Figure 2a), we present one of the possible pathways for a directional H–D exchange in an alternating CH_3_OH–D_2_O H-bonded network. Later, we provide further evidence for such a mechanism by modifying the initial D_2_O:CH_3_OH ratio (Figure 3). This mechanism is also supported by the previous TDS study of DePonte et al.^20^ for the H–D exchange between water and ethanol on Au(111), and the linear structure of the water–methanol network is in line with the AFM work of Voïtchovsky et al.^9^ Above the desorption temperature of methanol (Figure 2b), according to this exchange model, solely HDO should be produced. However, in this temperature range (140 K ≤ *T* < 160 K) the H–D exchange between water molecules is known to readily take place; thus, a mixture of HDO, H_2_O, and D_2_O is expected to form, while HDO is still the main isotopologue.

**Figure 2:**
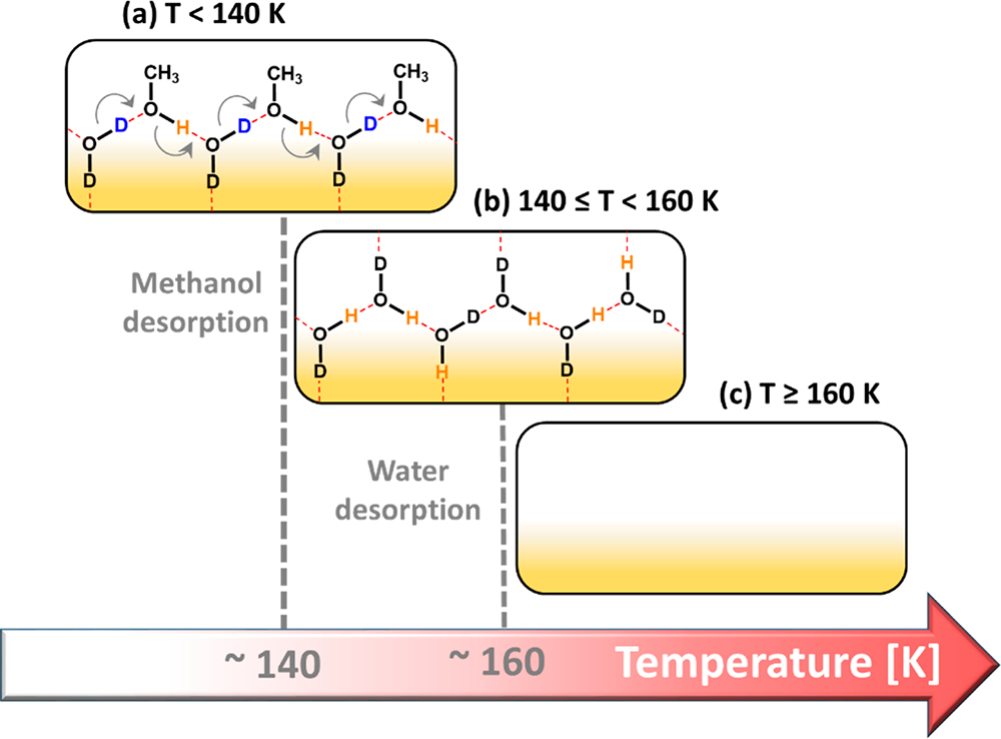
Schematic model of one of the possible structural evolution pathways of the adsorbed molecules with temperature following D_2_O and CH_3_OH coadsorption on the Cu(111) surface: (a) H-bonding and H–D exchange at low temperatures and (b) the remaining HDO product after methanol desorption. At this temperature range an H–D exchange between water molecules to form a mixture of HDO/D_2_O/H_2_O is possible. (c) Clean surface following water desorption (∼160 K). The D and H atoms which participate in the exchange are marked in blue and orange colors, respectively. The dashed red lines represent the H-bonds.

**Figure 3:**
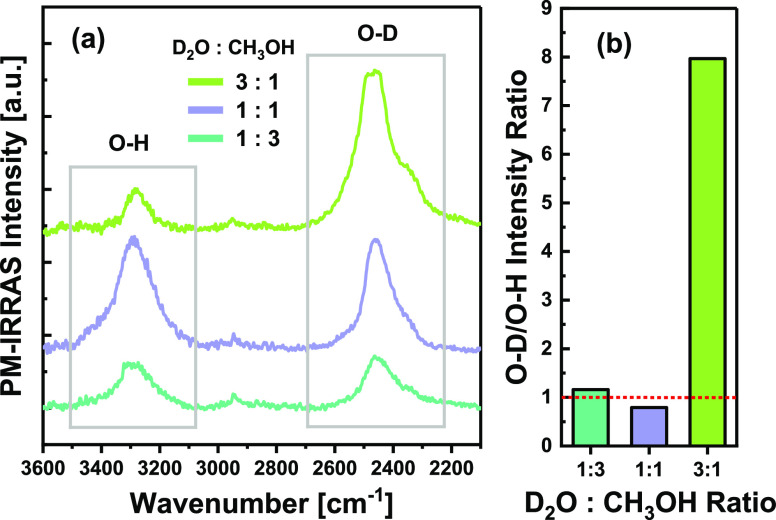
(a) PM-IRRAS spectra following the coadsorption of D_2_O and CH_3_OH with different initial ratios on Cu(111) at 95 K and subsequent heating to 140 K for methanol desorption. In all cases, the total dosage of D_2_O and CH_3_OH was 0.2 L. (b) Integrated intensities ratio of the O–D/O–H bands versus the initial D_2_O:CH_3_OH dosing ratio.

The exact temperature range in which the H–D exchange takes place can be inferred from comparing the evolution of the O–D band (2250–2750 cm^–1^) for D_2_O + CH_3_OH coadsorption (Figure 1b) with that of pure D_2_O adsorption (Figure 1a). As previously mentioned, for pure D_2_O adsorption the O–D stretching band remains unchanged with increasing the surface temperature up to 130 K (included), whereas for D_2_O + CH_3_OH adsorption some changes are observed in the H-bonded O–D band at 120–130 K. At 120 K, this band broadens and shifts to low wavenumbers, while at 130 K it splits into two peaks at 2447 and 2480 cm^–1^ with two shoulders at lower (with larger intensity) and higher (with smaller intensity) frequencies. As will be discussed later, we attribute these changes to the formation of methanol–OD (CH_3_OD) via H–D exchange. At the same temperature range, broadening of the O–H band, which originally belonged solely to CH_3_OH, is observed. This broadening can be associated with the contribution of HDO to the O–H band.

Another strong evidence for H–D exchange at 120–130 K can be obtained by careful examination of the methyl rocking modes ρ(CH_3_) in the PM-IRRAS spectra (Figure 1b). Methanol has two CH_3_ rocking modes, labeled as *a′* and *a″*, respectively (Table 1).^38^ At 95 K, the small peak at 1127 cm^–1^ is assigned to the *a′*-ρ(CH_3_), which is the dominant mode for CH_3_OH.^12,37^ The intensity of this peak decreases with temperature, and at 130 K it becomes undetectable, whereas an additional small peak at 1235 cm^–1^ emerges (Figure 1b, indicated by an arrow). This peak can be attributed to the *a″*-ρ(CH_3_) mode of CH_3_OD, for which the *a′*-ρ(CH_3_) mode is much weaker than the *a″*-ρ(CH_3_) mode.^38^ Overall, the changing rocking mode at 130 K suggests the conversion of CH_3_OH into CH_3_OD due to H–D exchange between D_2_O and CH_3_OH. It is also worth mentioning that the H–D exchange between methanol and water in thick films was found to take place at the same temperature range (120–130 K);^21^ thus, the Cu(111) surface has no significant catalytic effect on the exchange kinetics. Yet, as we showed in our previous study on methanol adsorption, the Cu surface orientation might affect the structure and ordering of the H-bonded clusters.^12^

Further supporting evidence for the possible exchange mechanism demonstrated in Figure 2 can be provided from PM-IRRAS measurements with different D_2_O:CH_3_OH initial ratios. Figure 3a shows the PM-IRRAS spectra obtained in three separate experiments in which different D_2_O:CH_3_OH ratios, with a total dosage of 0.2 L, were dosed onto the Cu(111) surface at 95 K and followed by heating to 140 K to desorb methanol. At this temperature, the relative intensities of the remaining O–H and O–D stretching bands can indicate the isotopic composition of the water isotopologues mixture (H_2_O, HDO, and D_2_O). An additional small peak is observed at 2945 cm^–1^, which is probably due to the C–H stretching of residual hydrocarbon contaminants.

The integrated intensity ratio of the O–D and O–H bands as a function of the initial D_2_O:CH_3_OH dosing ratio is presented in Figure 3b. The nearly equivalent intensities of the O–D and O–H bands for both 1:3 and 1:1 D_2_O:CH_3_OH ratios indicate that in both cases the exchange product is composed of ∼50% O–D groups and ∼50% O–H groups; i.e., half of the D atoms of D_2_O were exchanged with H atoms from CH_3_OH. A possible mechanism which might explain this isotopic composition, even when CH_3_OH is the major component (e.g., D_2_O:CH_3_OH of 1:3), is through the formation of HDO; i.e., if HDO is indeed the primary product of the H–D exchange between D_2_O and CH_3_OH, then an O–D/O–H ratio of ∼1 should be obtained after methanol desorption. This also fits the model presented in Figure 2 well. For an initial D_2_O:CH_3_OH dosing ratio of 3:1 (i.e., 0.15 L D_2_O + 0.05 L CH_3_OH, equivalent to 0.0375 and 0.01 ML, respectively) a much higher O–D/O–H ratio of ∼8 is obtained following methanol desorption (Figure 3b). In this case, the amount of the exchange product HDO is limited by the initial amount of CH_3_OH. Therefore, after the exchange a mixture of HDO and D_2_O is obtained, and since D_2_O is the major component, the shape of the O–D band is similar to that of pure D_2_O following crystallization. It should be noted that stoichiometrically, assuming a full conversion of CH_3_OH (0.01 ML) into CH_3_OD, the water product should be composed of 0.01 ML HDO and 0.0275 ML D_2_O, which yields an O–D/O–H ratio of 6.5. The higher O–D/O–H ratio (∼8) is either due to the enhanced intensity of the O–D band for crystalline D_2_O and/or slight inaccuracy in dosing the right amounts of CH_3_OH and D_2_O. Additionally, the intensity in PM-IRRAS is not directly proportional to the coverage of each bond as it depends on chemical and physical effects and is sensitive to the orientation of the bond with respect to the surface. In this case, since the O–H and O–D bonds are equivalent in terms of orientation and chemical environment, only physical effects, i.e., dynamic dipole coupling, should be taken into account. Dynamic dipole coupling might enhance the intensity of the O–D band compared to the O–H band while the O–D bonds are in excess (e.g., D_2_O:CH_3_OH of 3:1). However, the effect of dynamic dipole coupling on the O–D/O–H intensity ratio is expected to be negligible while this ratio is close to one (e.g., nearly equal coverage of O–D and O–H bonds).

In order to illustrate the contribution of the exchange product CH_3_OD to the 2200–2700 cm^–1^ band in Figure 1b, we performed a similar coadsorption experiment but with CH_3_OD instead of CH_3_OH (Figure 4). The frequencies of the observed peaks at 95 K and their assignments are summarized in Table 1. Similar to D_2_O + CH_3_OH coadsorption (Figure 1b), the addition of 0.1 L CH_3_OD to the preadsorbed 0.1 L D_2_O at 95 K causes the disappearance of the dangling O–D peak of D_2_O (∼2729 cm^–1^) accompanied by the appearance of the vibrational bands of CH_3_OD.^38^ These bands are marked with gray dashed lines in Figure 4a. In this case, the 2200–2700 cm^–1^ band is composed of the O–D stretching of CH_3_OD (2440 cm^–1^ at 95 K) and the overtone of the *a″*-ρ(CH_3_) mode (2476 cm^–1^ at 95 K) convoluted with the O–D band of D_2_O. At 130 K the shape of the 2200–2700 cm^–1^ band is similar to that obtained for D_2_O + CH_3_OH coadsorption at the same temperature, including the characteristic peaks of CH_3_OD at ∼2447 cm^–1^ and ∼2480 cm^–1^ (Figure 4b). This result corroborates our assignment of the newly formed peaks in the O–D band in Figure 1b (130 K) to the formation of CH_3_OD via the H–D exchange. Moreover, above the desorption temperature of methanol (*T* = 150 K), we can compare the O–D band to that of pure D_2_O adsorption (Figure 4c). The nearly identical triple-peak shape indicates that the methanol that was incorporated in the H-bonded structure has no significant effect on the crystallization of water once it desorbs. At this temperature a small peak at 3284 cm^–1^ is visible in the O–H stretching region (Figure 4a at 150 K, indicated by a gray arrow), which is probably due to H-impurities in the deuterated water. Nonetheless, the fact that only a minor peak is observed in the O–H region confirms that the O–H band in the D_2_O + CH_3_OH coadsorption experiment (Figure 1b) after methanol desorption (140–150 K) has originated from the exchange with CH_3_OH, and is not an artifact due to exchange with residual H_2_ or H_2_O in the measurement chamber.

**Figure 4:**
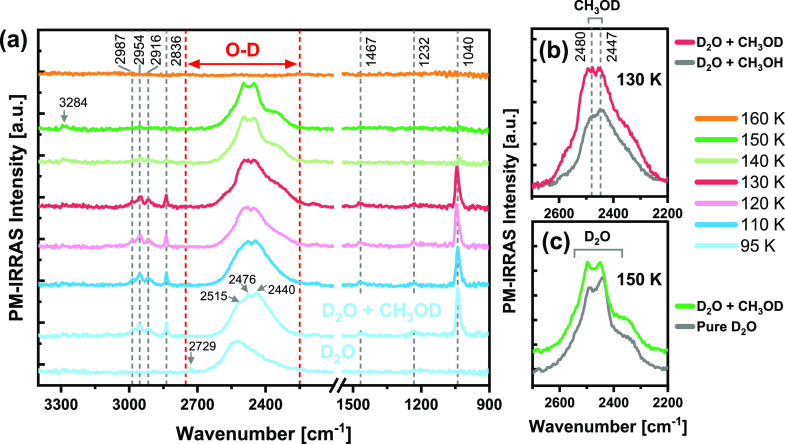
(a) PM-IRRAS spectra of 0.1 L D_2_O + 0.1 L CH_3_OD coadsorption on the Cu(111) surface, followed by annealing to different temperatures up to 160 K. Plots on the right-hand side show comparisons between the O–D band of D_2_O coadsorption with CH_3_OD and CH_3_OH at 130 K in part b and of pure D_2_O and D_2_O + CH_3_OD coadsorption at 150 K in part c. Gray dashed lines indicate the vibrational bands of CH_3_OD. Detailed assignments of the peaks are listed in Table 1.

The phenomenon of hydrogen exchange between methanol and water in the adsorbed state is not limited to the case of methanol interaction with preadsorbed ASW. In the Supporting Information, we demonstrate that the H–D exchange also takes place both in the case of opposite dosing order (i.e., when CH_3_OH is dosed prior to D_2_O) and when CH_3_OH is dosed onto a surface covered with a crystalline layer of D_2_O rather than amorphous.

In conclusion, our results provide new insights into the nature of the H-bonding between water and methanol in the adsorbed state and how the temperature and the initial water/methanol composition affect hydrogen exchange. The first interaction between methanol and preadsorbed D_2_O at 95 K is through H-bonding with the dangling OD groups of the uncoordinated D_2_O molecules of the amorphous clusters. At higher temperatures, and particularly above 120 K, H–D exchange takes place between the coadsorbed D_2_O and CH_3_OH molecules to form HDO and CH_3_OD. Between the desorption temperatures of methanol (∼140 K) and water (∼160 K), a mixture of hydrogen-related water isotopologues remains on the surface. The dependence of the exchange ratio on the initial D_2_O/CH_3_OH composition supports an exchange mechanism through an alternating H-bonded network of D_2_O and CH_3_OH. Although being a short-lived molecular precursor state in the MSR reaction, this alternating H-bonded network might dictate short intermolecular distances between the reactants.

## Methods

The PM-IRRAS experiments were performed in a dedicated UHV system which is described in detail in our previous works.^3,12,40^ The base pressures in the preparation and measurement chambers were 2–5 × 10^–10^ mbar and ∼1 × 10^–10^ mbar, respectively. To clean the Cu(111) single crystal, 2–3 cycles of Ar^+^ sputtering (5 × 10^–6^ mbar, 1 kV, 20–30 min) and annealing (10 min at 773 K) were performed before each experiment. Vapors of D_2_O, CH_3_OH, and CH_3_OD were dosed separately into the measurement chamber through leak valves after the sample was cooled to 95 K. Before dosing, both the methanol and water liquid reservoirs were outgassed by several freeze–pump–thaw cycles. While dosing, the pressure reading of the hot cathode ionization gauge was corrected using the corresponding gas correction factor for water (1.12) and methanol (1.85). PM-IRRAS spectra were measured prior to dosing in UHV and following water and/or methanol adsorption at 95 K and gradual heating to 160 K. We used maximum dephasing frequencies (a setting of the photoelastic modulator) of 1000 and 2600 cm^–1^ to cover the spectral range of interest and averaged 600 scans for each measurement. All the PM-IRRAS spectra presented here were baseline corrected by subtracting a reference spectra measured in UHV before each experiment, which removed the second-order Bessel function.

## Supporting Information Available

Experimental and characterization information (PDF)
Transparent Peer Review report available (PDF)
