# Supplementary material for: Hydrogen Exchange through Hydrogen Bonding between Methanol and Water in the Adsorbed State on Cu(111)
Source: J Phys Chem Lett. 2023 Mar 8;14(10):2644–50. doi: 10.1021/acs.jpclett.3c00161 (PMC10026171; doi:10.1021/acs.jpclett.3c00161)
Supplement: Supplementary file 1 — jz3c00161_si_001.pdf [file jz3c00161_si_001.pdf]

Supporting Information for

Hydrogen Exchange through Hydrogen Bonding between Methanol and Water in the Adsorbed State on Cu(111)

Roey Ben David,[†], Adva Ben Yaacov, [†], Baran Eren,*,[†]

†*Department of Chemical and Biological Physics, Weizmann Institute of Science, 234 Herzl Street, 76100 Rehovot, Israel,*

* E-mail: baran.eren@weizmann.ac.il Phone: +972 8-934-3708

Section S1: $CH_3OH + D_2O$ co-adsorption on Cu(111): Effect of dosing order

Figure S1 shows the evolution of the PM-IRRAS spectra with temperature following the co-adsorption of CH_3OH and D_2O on Cu(111) (0.1 L of each, dosed at 95 K). Unlike the procedure presented in the main text, here CH_3OH was dosed prior to D_2O. The appearance of both O-H and O-D bands following methanol desorption (T = 140-150 K in Figure S1) provides a clear evidence for the H-D exchange between D_2O and CH_3OH as discussed in the paper. A detailed assignment of the PM-IRRAS peaks to the vibrational bands of methanol and water can be found in Table 1 in the paper.

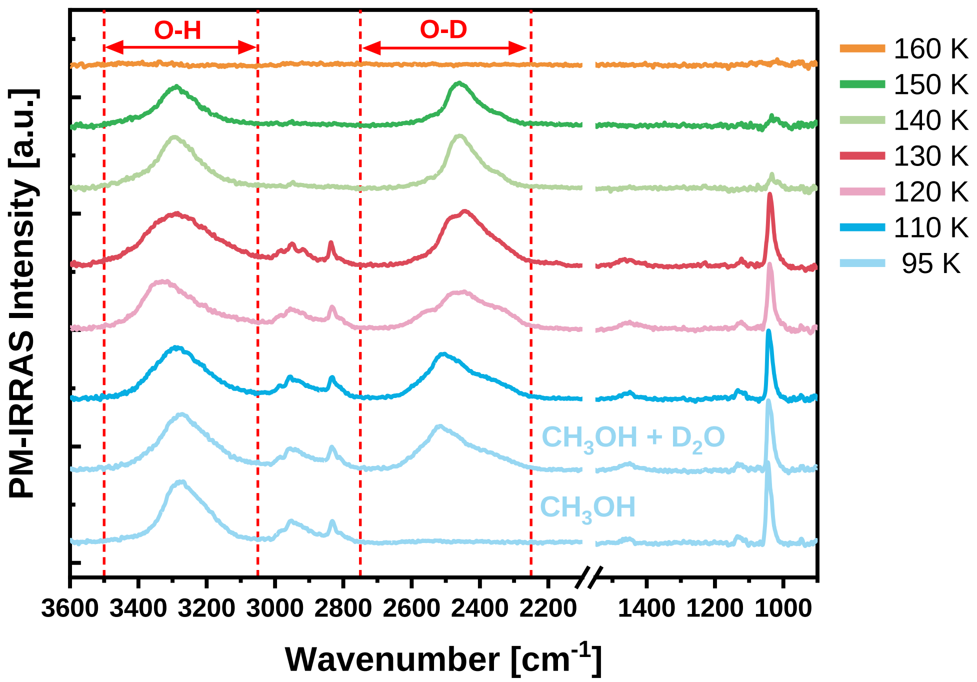

Figure S1. PM-IRRAS spectra of CH_3OH + D_2O (0.1 L each) co-adsorption on Cu(111). In this experiment, CH_3OH was dosed *before* D_2O at 95 K followed by gradual heating the surface up to 160 K. Dashed red lines indicate the O-H and O-D vibrational bands.

Section S2: CH_3OH interaction with a crystalline (ordered) D_2O layer on Cu(111)

In the co-adsorption experiments described in the main text, D_2O was dosed prior to methanol (CH_3OH or CH_3OD) on a cooled Cu(111) surface at 95 K. Under these conditions, the adsorbed D_2O molecules form amorphous clusters (i.e., amorphous solid water, ASW) on the Cu(111) surface. Hence, the initial interaction of methanol (dosed at 95 K) is with an amorphous phase of D_2O. This phase is metastable[1] and contains more dangling (non-hydrogen-bonded) O-D groups compared to the ordered (crystalline) phase with the well-known double bilayer structure[2,3]. Hence, the thermodynamic driving force to form H-bonded structures of D_2O and CH_3OH, which allow the H-D exchange through the H-bonds, is expected to be larger for an initial state of ASW compared to the ordered phase.

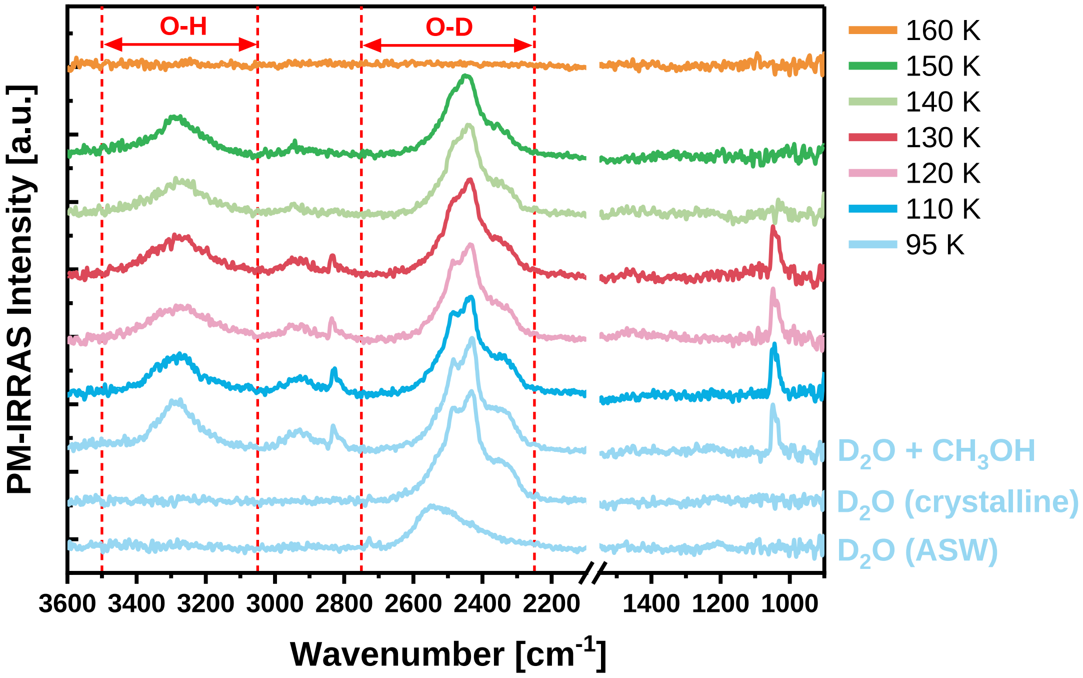

Figure S2. PM-IRRAS spectra of D_2O (ASW and crystalline) adsorption and co-adsorption of crystalline D_2O with CH_3OH on a Cu(111) at 95 K. After CH_3OH addition to the crystalline D_2O layer the surface was gradually heated to 160 K. The crystalline layer of D_2O was prepared by annealing the ASW layer at 145 K and cooling back to 95 K. Dashed red lines indicate the O-H and O-D vibrational bands of the adsorbed species.

In order to examine whether H-D exchange also occurs between CH_3OH and crystalline (ordered) layer of D_2O, we performed a co-adsorption experiment starting with a crystalline D_2O layer *before* CH_3OH dosing. We produced this layer by annealing the ASW layer of D_2O (deposited at 95 K) at 145 K, i.e., above the onset temperature for crystallization[2,4], and cooling back to 95 K. Figure S2 shows the PM-IRRAS spectra of the initial D_2O layer (ASW), formed by dosing 0.1 L of D_2O at 95 K, and the crystalline (ordered) D_2O layer followed by CH_3OH adsorption (0.1 L at 95 K) and gradual heating up to 160 K. The interpretation of the shape of the O-D band for ASW and crystalline D_2O is discussed in the main text. The changes in the O-D and O-H bands with temperature, particularly at 120-130 K, and the remaining O-H band after methanol desorption (140 K $\leq$ T < 160 K) confirm the occurrence of H-D exchange between crystalline D_2O and CH_3OH. Note that the lower intensity of the O-H band compared to the O-D band at 140-150 K is due to initially low CH_3OH coverage (inaccurate CH_3OH dosing).

References

(1) Smith, R. S.; Matthiesen, J.; Knox, J.; Kay, B. D. Crystallization Kinetics and Excess Free Energy of H_2O and D_2O Nanoscale Films of Amorphous Solid Water. *J. Phys. Chem. A* **2011**, *115* (23), 5908–5917. https://doi.org/10.1021/jp110297q.

(2) Mehlhorn, M.; Morgenstern, K. Faceting during the Transformation of Amorphous to Crystalline Ice. *Phys. Rev. Lett.* **2007**, *99* (24), 5–8. https://doi.org/10.1103/PhysRevLett.99.246101.

(3) Stacchiola, D.; Park, J. B.; Liu, P.; Ma, S.; Yang, F.; Starr, D. E.; Muller, E.; Sutter, P.; Hrbek, J. Water Nucleation on Gold: Existence of a Unique Double Bilayer. *J. Phys. Chem. C* **2009**, *113* (34), 15102–15105. https://doi.org/10.1021/jp904875h.

(4) Backus, E. H. G.; Grecea, M. L.; Kleyn, A. W.; Bonn, M. Surface Crystallization of Amorphous Solid Water. *Phys. Rev. Lett.* **2004**, *92* (23), 1–4. https://doi.org/10.1103/PhysRevLett.92.236101.
