# Supplementary material for: Hydrogen Exchange through Hydrogen Bonding between Methanol and Water in the Adsorbed State on Cu(111)
Source: J Phys Chem Lett. 2023 Mar 8;14(10):2644–50. doi: 10.1021/acs.jpclett.3c00161 (PMC10026171; doi:10.1021/acs.jpclett.3c00161)
Supplement: Supplementary file 2 — jz3c00161_si_002.pdf [file jz3c00161_si_002.pdf]

jz-2023-00161j.R1

Name: Peer Review Information for "Hydrogen Exchange Through Hydrogen Bonding Between Methanol and Water in the Adsorbed State on Cu(111)"

First Round of Reviewer Comments

Reviewer: 1

Comments to the Author

1. What is the major advance reported in the paper?

Accurate measurements of H D exchange between methanol and water on a well-defined Cu(111) surface are made in which the hydrogen bonding between molecules and proton hoping can be quantified with temperature controlled IR measurements in situ.

2. What is the immediate significance of this advance?

Experimental data like these provide good benchmarks for ab initio calculations of these H transfer events, which are ubiquitous in chemistry and biology.

3. Technical suggestions

This is a high-quality study and a well written paper. Given that this data will probably be modeled by other groups it is important that the measurements be as well calibrated as possible. Specifically, units of Langmuir are meaningless as a way to quantify surface coverage as different molecules have different ionization cross sections and fragmentation patterns. On page 6 equal Langmuir amounts of MeOH and H2O are deposited – this should be quantified in monolayers of each after their ionization cross sections and fragmentation patterns are accounted for. Page 10 talks about desorption of 0.05 L of HDO etc. This should be given in monolayers or molecules per cm2. Also, on page 7 – is it fair to compare the intensities of the O-H and O-D stretches as a way to quantify the number of molecules the exchange has occurred in? Dipole coupling effects in IR spectroscopy can make this tricky in many systems so this should be expanded upon.

Is there any justification of the chain model on page 8, Figure 2? Be clear on this in the paper as it will be the first model that is use in simulations by others.

Reviewer: 2

Comments to the Author

This is a thorough study of hydrogen exchange that occurs due to the co-adsorption of methanol and water, with this film supported on an underlying Cu(111) substrate. The authors present good evidence that isotope exchange occurs between these co-adsorbed species. This is accomplished by viewing IR spectra that focus on the OH/OD signatures for various species in the exchange medium. My first point is therefore that the demonstration of isotope exchange in sequential temperature regimes seems quite convincing. My second point focuses on what is missing: The authors use their data to propose an exchange mechanism that involves hoping between alternating methanol and water molecules in a hydrogen-bonded network. While this is reasonable, the point is not proven. It would have been informative if the authors had also pre-formed crystalline ice and then dosed the methanol. This would create a different surface presentation to help test the proposed mechanism. If the authors do this, or have done this, I would support JPC/L publication without any further inquiries. While this would require some more data to be presented (not just discussed), it would further enrich the Letter. The Letter now is fine, but the suggested addition would enhance its utility to the community.

Author's Response to Peer Review Comments:

27-Feb-2023

Dear Prof. Editor

Journal of Physical Chemistry Letters,

Re: Manuscript No. jz-2023-00161j

Thank you for sending us the reviewers' comments regarding our manuscript: **"Hydrogen Exchange through Hydrogen Bonding between Methanol and Water in the Adsorbed State on Cu(111)"** submitted for consideration in the *Journal of Physical Chemistry Letters*.

We have revised the manuscript in full accordance with the reviewers' comments. For your convenience, all essential corrections and additions in the revised manuscript are marked in red.

We would like to sincerely thank the reviewers for their insightful comments, which helped us to improve our manuscript.

We hereby provide an explicit answer to each comment of the reviewers.

---

## Reviewer #1

*Comments:*

*1. What is the major advance reported in the paper?*

*Accurate measurements of H-D exchange between methanol and water on a well-defined Cu(111) surface are made in which the hydrogen bonding between molecules and proton hoping can be quantified with temperature controlled IR measurements in situ.*

*2. What is the immediate significance of this advance?*

*Experimental data like these provide good benchmarks for ab initio calculations of these H transfer events, which are ubiquitous in chemistry and biology.*

We would like to thank the reviewer for their comments. We are happy to hear that the reviewer finds our experimental study accurate and valuable for future theoretical studies.

**Comment 1**

*Technical suggestions: This is a high-quality study and a well written paper. Given that this data will probably be modeled by other groups it is important that the measurements be as well calibrated as possible. Specifically, units of Langmuir are meaningless as a way to quantify surface coverage as different molecules have different ionization cross sections and fragmentation patterns. On page 6 equal Langmuir amounts of MeOH and $D_2O$ are deposited – this should be quantified in monolayers of each after their ionization cross sections and fragmentation patterns are accounted for. Page 10 talks about desorption of 0.05 L of HDO etc. This should be given in monolayers or molecules per $cm^2$.*

**Answer to comment 1**

We added the calculated surface coverages (in monolayers) of water/methanol in the relevant parts in the text. The calculation is based on the kinetic theory of gases, assuming a sticking coefficient of ~1 at 95 K (the dosing temperature) and using the theoretical atomic density of the Cu(111) surface ($1.77\times10^{19}$ atoms/$m^2$). We mentioned the evaluated coverages in the beginning of the results description:

*"In both cases, 0.1 Langmuirs (1 L $=10^{-6}$ Torr·s) of each gas were dosed at 95 K, and $D_2O$ was dosed prior to methanol in the case of $D_2O$ + $CH_3OH$. Using the kinetic theory of gases and assuming the sticking coefficient to be unity, exposure of 0.1 L is equivalent to a coverage of 0.02 monolayer (ML) of $CH_3OH$ and 0.025 ML of $D_2O$."*

We also used the calculated coverages for our stoichiometric calculations:

*" For an initial $D_2O$:$CH_3OH$ dosing ratio of 3:1 (i.e., 0.15 L $D_2O$ + 0.05 L $CH_3OH$, equivalent to 0.0375 ML and 0.01 ML, respectively) a much higher O-D/O-H ratio of ~8 is obtained following methanol desorption (Figure 3b). In this case, the amount of the exchange product HDO is limited by the initial amount of $CH_3OH$. Therefore, after the exchange a mixture of HDO and $D_2O$ is obtained, and since $D_2O$ is the major component the shape of the O-D band is similar to that of pure $D_2O$ following crystallization. It should be noted that stoichiometrically, assuming a full conversion of $CH_3OH$ (0.01 ML) into $CH_3OD$, the water product should be composed of 0.01 ML HDO and 0.0275 ML $D_2O$, which yields an O-D/O-H ratio of 6.5."*

It is worth noting that for both dosage (in Langmuir) and coverage (in ML) calculations the dosing pressure, measured by the hot cathode ionization gauge (calibrated for $N_2$), was corrected using the corresponding gas correction factors for methanol (1.85) and water (1.12). The gas correction factors take into account the different ionization cross section and fragmentation patterns. We mentioned this technical issue in the methods section:

*"While dosing, the pressure reading of the hot cathode ionization gauge was corrected using the corresponding gas correction factor for water (1.12) and methanol (1.85)."*

**Comment 2**

*Also, on page 7 – is it fair to compare the intensities of the O-H and O-D stretches as a way to quantify the number of molecules the exchange has occurred in? Dipole coupling effects in IR spectroscopy can make this tricky in many systems so this should be expanded upon.*

**Answer to comment 2**

Using the integrated PM-IRRAS intensities of the O-H and O-D stretching bands as a measure for the coverage of the O-H and O-D bonds in the water mixture following the exchange is indeed not straightforward. Here, we used the intensity ratio of the O-D and O-H bands as a measure for the extent of exchange. This intensity ratio may deviate from the actual (true) ratio of the number of O-D and O-H bonds due to several factors which affect the intensity in PM-IRRAS. We added explanation about the expected effect of these factors, including dynamic dipole coupling:

*"Additionally, the intensity in PM-IRRAS is not directly proportional to the coverage of each bond as it depends on chemical and physical effects and is sensitive to the orientation of the bond with respect to the surface. In this case, since the O-H and O-D bonds are equivalent in terms of orientation and chemical environment, only physical effects, i.e., dynamic dipole coupling, should be taken into account. Dynamic dipole coupling might enhance the intensity of the O-D band compared to the O-H band while the O-D bonds are in excess (e.g., $D_2O$:$CH_3OH$ of 3:1). However, the effect of dynamic dipole coupling on the O-D/O-H intensity ratio is expected to be negligible while this ratio is close to one (e.g., nearly equal coverage of O-D and O-H bonds)."*

**Comment 3**

*Is there any justification of the chain model on page 8, Figure 2? Be clear on this in the paper as it will be the first model that is use in simulations by others.*

**Answer to comment 3**

Our justification for the exchange mechanism through a chain-like H-bonded structures of alternating $CH_3OH$ and $D_2O$ molecules is given by the PM-IRRAS experiments with different initial $CH_3OH$:$D_2O$ ratios (Figure 3 in the manuscript). Moreover, this model is supported by previous studies of water-ethanol adsorption on Au(111) and water-methanol interface with graphene. We tried to emphasize these points in the following sentences:

*"In this model for $T < 140$ K (Figure 2a), we present one of the possible pathways for a directional H-D exchange in an alternating $CH_3OH$-$D_2O$ H-bonded network. Later, we provide further evidence for such a mechanism by modifying the initial $D_2O$:$CH_3OH$ ratio (Figure 3). This mechanism is also supported by the previous TDS study of DePonte et al.[20] for the H-D exchange between water and ethanol on Au(111), and the linear structure of the water-methanol network is in line with the AFM work of Voïtchovsky et al.[9]."*

## Reviewer #2

*Comments: This is a thorough study of hydrogen exchange that occurs due to the co-adsorption of methanol and water, with this film supported on an underlying Cu(111) substrate. The authors present good evidence that isotope exchange occurs between these co-adsorbed species. This is accomplished by viewing IR spectra that focus on the OH/OD signatures for various species in the exchange medium. My first point is therefore that the demonstration of isotope exchange in sequential temperature regimes seems quite convincing.*

We would like to thank the reviewer for their comments. We are pleased to hear that the reviewer finds our results and proposed mechanism convincing.

**Comment 1**

*My second point focuses on what is missing: The authors use their data to propose an exchange mechanism that involves hoping between alternating methanol and water molecules in a hydrogen-bonded network. While this is reasonable, the point is not proven. It would have been informative if the authors had also pre-formed crystalline ice and then dosed the methanol. This would create a different surface presentation to help test the proposed mechanism. If the authors do this, or have done this, I would support JPC/L publication without any further inquiries. While this would require some more data to be presented (not just discussed), it would further enrich the Letter. The Letter now is fine, but the suggested addition would enhance its utility to the community.*

**Answer to comment 1**

Indeed, the results presented in the manuscript refers to methanol ($CH_3OH$ or $CH_3OD$) interaction with pre-adsorbed amorphous solid water (ASW) of $D_2O$, which is the phase that obtained at low surface temperatures (95 K). The idea of studying the interaction of methanol with a crystalline (ordered) layer of $D_2O$ is very interesting since this phase (presumably a double bilayer) is expected to be more stable than the amorphous phase which is clearly metastable. Hence, following this important comment, we performed an additional experiment of $CH_3OH$ adsorption on a crystalline $D_2O$ layer on a Cu(111). We produced this layer by annealing the ASW phase (deposited at 95 K) above the crystallization onset and cooling back to 95 K before $CH_3OH$ dosing. The results of this experiment are presented and discussed in the Supporting Information (see Section S2) we added. The results clearly demonstrate the occurrence of H-D exchange between $CH_3OH$ and crystalline $D_2O$, as both O-H and O-D bands remain in the PM-IRRAS spectra following methanol desorption (T = 140-150 K). Moreover, we examined the case of opposite dosing ordered, i.e., while $CH_3OH$ is dosed before $D_2O$, which gives another surface presentation that supports and generalizes the H-D exchange mechanism (Section S1 in the Supporting Information).

We also referred to the new results in the main text:

*"The phenomenon of hydrogen exchange between methanol and water in the adsorbed state is not limited to the case of methanol interaction with pre-adsorbed ASW. In the Supporting Information (SI) we demonstrate that the H-D exchange also takes place both in the case of opposite dosing order (i.e., when $CH_3OH$ is dosed prior to $D_2O$) and when $CH_3OH$ is co-adsorbed onto a surface covered with a crystalline layer of $D_2O$ rather than amorphous."*
